# Development of a Salutogenesis Workshop for SPPs to Help Them, Their Athletes, and the Athlete’s Entourage Better Cope With Uncertainty During the COVID-19 Pandemic

**DOI:** 10.3389/fpsyg.2021.612264

**Published:** 2021-05-21

**Authors:** Sascha Leisterer, Franziska Lautenbach, Nadja Walter, Lara Kronenberg, Anne-Marie Elbe

**Affiliations:** ^1^Department of Sport Psychology, Institute of Sport Psychology and Sport Pedagogy, Faculty of Sports Science, Leipzig University, Leipzig, Germany; ^2^Sport Psychology, Department of Sport Sciences, Faculty of Humanities and Social Sciences, Humboldt-Universität zu Berlin, Berlin, Germany

**Keywords:** mental health, resilience, staff, stress, psychological counseling

## Abstract

The COVID-19 pandemic is also called a crisis of uncertainty because of so many unforeseeable events like canceled qualification competitions, loss of training facilities, and postponement of the Olympic games. Athletes and their entourage experience this uncertainty as stressful. Sport psychology practitioners (SPPs) are in a key position to support athletes in coping with these unforeseeable stressors. However, SPPs are similarly affected by the COVID-19 pandemic and simultaneously have to cope with stress. Salutogenesis, which describes how to manage stress and to stay well, provides a theoretical approach to how to cope with uncertainty. The salutogenetic approach aims at strengthening individuals’ sense of coherence (SoC) and consists of three components, namely comprehensibility, manageability, and meaningfulness. Although it is known that the SoC can be enhanced *via* psychological skills training, so far, this approach has not been systematically applied to the elite sport context. Athletes have been advised to see SPPs for help; thus, the question of how SPPs handle the time of uncertainty while supporting others emerges. The aim of this contribution was to outline how the salutogenetic approach can be applied to strengthening SPPs’ SoC *via* a single-day four-part workshop. Additionally, we applied the workshop to *N* = 26 volleyball coaches and evaluated the workshop’s effects on participants’ psychological aspects [i.e., the Sense of Coherence—Leipziger short version (SoC-L9), resilience (RS-13): coping with uncertainty, affective response, and stress *via* semantic differentials] and the workshop’s quality ratings (i.e., Quality Questionnaire for Sport Psychological Coaching, QS-17). The evaluation provides results that show a positive impact on a descriptive level of the participants’ SoC, uncertainty, affect, and stress perception; however, the results show no significant main effect of time [*F*(8, 10) = 1.04, *p* = 0.467, η_*p*_^2^ = 0.454]. Workshop quality (on average, 3.60 ± 0.35 out of 4.00) and skill acquisition (on average, 3.00 ± 0.64 out of 4.00) were positively evaluated; 82.00% of the participants would use the learned tools in the future. Thus, we outline how this workshop might help strengthen SPPs’ SoC and at the same time empower them to strengthen their athletes’ SoC. Overall, we add a theoretical (i.e., salutogenesis in sports) and a practical perspective (i.e., coping techniques based on salutogenesis) on how to cope with the COVID-19 pandemic for SPPS, athletes, and their support network.

## Introduction

For the first time in the history of the modern Olympic Games, the Olympics were postponed from 2020 to 2021 due to the COVID-19 pandemic. The regular 4-year cycle was interrupted and impacted the systematic planning, controlling, and performing at a top level in elite sports. No one had anticipated that an international health crisis would have such tremendous effects on the athletes and their entourage, such as the postponement of the Olympic Games, changes in scheduled qualification competitions, or limited training possibilities. The athlete’s support network is a combination of different stakeholders who provide support for peak performances in sports. Athletes play a central role in this network because they are the performers; however, they are surrounded by a large staff network [e.g., coaches and sport psychology practitioners (SPP)] who support them in achieving their peak performance. During COVID-19, it has become even more important to support athletes and especially also to reduce the effects of uncertainty. This support can, for example, help foster athletes’ identities and help manage their careers (Andreato et al., 2020). SPPs are crucial to how the athletes and their support network deal with the unforeseen effects of COVID-19, which can also be called a crisis of uncertainty (Andreato et al., 2020). Studies have already shown an increase in perceived stress and in dysfunctional psychobiosocial states (Di Fronso et al., 2020). Further expected negative mental reactions are anxiety, depression, and maladaptive behavior (Andreato et al., 2020). This uncertainty can have an immense impact on athletes’ careers, such as problems with investing the effort to continue their training, the pausing of all routines, or the retirement from sports (Samuel et al., 2020; Stambulova et al., 2020). One important function of SPPs is to keep athletes on track for the Olympic Games 2021 and to support their mental well-being during this crisis of uncertainty by planning ahead despite all obstacles they might encounter due to the current global health crisis (e.g., Papaioannou et al., 2020).

In peak performance sports, the athlete’s support network tries to anticipate and plan everything as intricately as possible, e.g., with training logs, season planning, specialized nutrition, etc. However, the COVID-19 pandemic shows that very little can be planned or controlled, which increases the vulnerability of athletes’ physical and mental well-being (Taku and Arai, 2020; Mehrsafar et al., 2020). Athletes may feel lost or stuck since they have limited access to training facilities and competitions, which are the center of their daily lives as sportswomen and sportsmen. This negative effect especially on athletes with a highly developed athletic identity who show cognitive and emotional maladaptations (Costa et al., 2020) can also be seen in similar situations like injuries (Spielmann et al., 2019), changes in rules (Lobinger et al., 2010), or boycotts of important competitions (Crossman and Lappage, 2002). It is important for athletes to successfully get through injury rehabilitation, to quickly adapt to rule changes, or to feel supported when one is not permitted to participate in the most important competition of one’s life. The athlete’s support network and especially SPPs are in charge of supporting athletes to deal with these situations (Lim and Pranata, 2020). SPPs—as counselors for athletes who are normally not directly involved in the athlete’s situation as such—generally support athletes in coping with challenging situations. Yet, SPPs are simultaneously affected by the COVID-19 pandemic. The entire athlete’s support network has to struggle with the unforeseeable during the next year of preparation for the Olympic Games in 2021. The question therefore arises of how to best cope with this situation of uncertainty.

SPPs play an important role in the athlete’s support network to improve the mental health of others—mainly athletes (Henriksen et al., 2020). The question, therefore, arises asking how SPPs can support athletes if they are struggling with uncertainty. The salutogenetic approach could be one avenue to answering this question, as it focuses on building up inner resources against unforeseen obstacles (Antonovsky, 1993b). Salutogenesis is an approach that explains which factors favor mental and physical health, in contrast to factors that cause disease. Salutogenesis relates health, stress, and coping. We argue that helping SPPs to strengthen their inner resources to overcome the unforeseeable of the COVID-19 pandemic will support them in empowering others, such as athletes, to stay on track for the Olympic Games 2021. We, therefore, propose and describe a workshop for SPPs aimed at developing specific coping techniques to teach athletes how to cope with the unforeseeable. At the same time, this workshop is also an intervention for SPPs to enhance their inner resources against the unforeseeable.

## Theory and Empirical Data on Salutogenesis

In salutogenesis, stressors, such as unforeseen events, might imbalance the continuum of well-being and ill-being (Antonovsky, 1993a,b). Individuals’ well-being can be secured *via* effectively coping with such stressors. Successful coping strategies can be derived from the sense of coherence (SoC), a central element of the salutogenetic approach.

**FIGURE 1:**
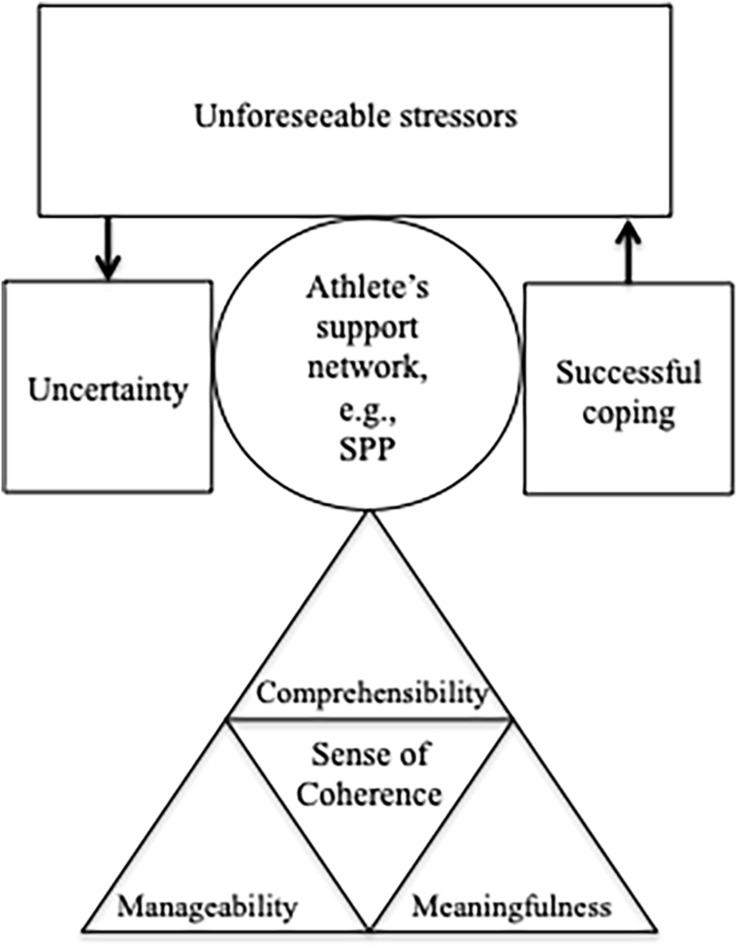
The salutogenesis model: the sense of coherence helps the individual to balance the stress caused by uncertainty *via* successful coping strategies (own depiction).

The SoC defines an individuals’ world view as an extensive but flexible confidence to cope with stressors. In other words, an individual with a high SoC is confident that life itself makes sense, is good, and that even though challenges and problems occur, they can be handled. The SoC consists of three components (Antonovsky, 1993b): comprehensibility, manageability, and meaningfulness (in Figure 1, depicted as triangles forming the SoC). Comprehensibility means that individuals can integrate the perceived stressor into their individual world view as an explicable, structured, and predictable stimulus (Eriksson, 2017). Comprehensibility refers to the cognitive component of the SoC and helps in managing stressors (Hochwälder, 2019). It encompasses being able to identify stressors correctly and choosing an appropriate coping strategy. If comprehensibility is well developed in an individual, the individual is able to encounter stressors with suitable coping strategies in a predictable way. Thus, individuals with high comprehensibility know and understand how to manage stressors. Manageability refers to the behavioral component of the SoC and describes the belief in individually realizable coping skills (Hochwälder, 2019). It relates to a feeling of self-efficacy and illustrates a feeling of trust in one’s coping skills to effectively manage stressors. Here, individuals rely on resources to cope with a stressor and trust in themselves to use helpful resources adequately in order to control upcoming stressors (Eriksson and Mittelmark, 2017; Mittelmark et al., 2017). Meaningfulness describes a motivational–emotional drive to actively deal with stressors as meaningful challenges in one’s life with the adaptation of manageable coping strategies (Hochwälder, 2019). It supports individuals in using coping strategies to recognize stressors as meaningful challenges. In doing so, coping with these challenges leads to a feeling of satisfaction and increased confidence in the ability to take care of oneself.

Individuals that have high comprehensibility, manageability, and meaningfulness are very resourceful in seeking out coping strategies. Such strategies could include seeking out valid news sources (comprehensibility), employing successful time management (manageability), or feeling social belonging and support (meaningfulness). The stronger the SoC, the more likely it is to stay resilient (Eriksson and Lindström, 2005).

During the COVID-19 pandemic, athletes are confronted with different concrete stressors that they need to cope with. A strong SoC supports athletes and their entourage in order to maintain equilibrium. This equilibrium can be visualized as a scale that ranges from uncertainty due to unforeseeable stressors to successful coping (see Figure 1). Among these are the unforeseen postponement of the Olympics, unforeseeable competition procedures, or unforeseen questions that arise concerning athletes’ ongoing careers (see more detailed stressors in Lautenbach et al., 2021). The sum of stressors can lead to an imbalance in the ill- and well-being continuum, which is described as the crisis of uncertainty (Figure 1). According to the salutogenetic approach, it is easier for individuals who have a well-developed SoC to cope with these stressors. Yet, to our knowledge, there is currently no explicit salutogenetic approach that is applied in sport psychology even if selected strategies exist and might already be implicitly taught.

In these times, however, it seems difficult to assess uncertainty as a comprehensive, manageable stressor that can be accepted as a meaningful challenge by athletes and their entourage. An uncertain future infiltrates comprehensibility, manageability, and meaningfulness to various extents: the uncertainty regarding the effects of the COVID-19 pandemic on the world of sports seems to be incomprehensible since its determinants and consequences are unpredictable. By that, it is difficult to find a structure of uncertainty when its determinants remain unclear or when knowledge about COVID-19 changes rapidly due to the ongoing publication of new research results. Finally, uncertainty seems to be unpredictable *per se* as no one knows what might happen next. Thus, the question arises, what athletes and their entourage can do to satisfactorily understand and appropriately predict the current pandemic situation, which is accompanied by uncertainty to deal with the current uncertain situation in sports. It is difficult, but not impossible, to manage this time of uncertainty. For example, athletes face an entirely new interruption of their preparations for the 2020 Olympics due to the pandemic situation, but still, many athletes know how to manage similar interruptions (e.g., injuries) to come back again. Last but not least, uncertainty can force athletes and their entourage to question whether it is meaningful to cope with the current situation when the future of training and competition is rather vague (i.e., “Why prepare for the Olympics if it is unclear whether they can take place in 2021?”). However, salutogenesis provides a suitable approach in sport psychology practice on how to use psychological skills to empower athletes and their support network in order to cope with uncertainty as a critical time in an athletic career.

Especially in critical times of an athletic career, research shows that a strong SoC is associated with many benefits. Generally, a higher SoC in elite athletes correlates with more positively developed mental skills (Fallby et al., 2006) and higher subjective well-being (Mayer and Thiel, 2014). Comparisons between students who are regularly physically active and those who are not indicate that regular physical activity enhances individuals’ SoC and lowers mental disturbances (Jindo et al., 2018). This relation between being active and an enhanced SoC seems to be more intense when individuals participate regularly in successful competitive sports (Jindo et al., 2018). Additionally, a stronger SoC seems to prevent competitive athletes from displaying morally questionable behavior, such as doping (Sheykhangafshe et al., 2020). However, athletes’ SoC can vary throughout their careers. Unforeseen negative experiences like injuries, which lead to a long recovery break or even to a career termination, challenge athletes’ SoC (Mayer and Thiel, 2014). Taking into account that a high SoC has many benefits but may be dynamic, it is important to ask how the SoC can be strengthened.

Overall, although the SoC is seen as a stable construct, research indicates that highly stressful events such as the COVID-19 pandemic may decrease the SoC, as has been shown for other destabilizing life situations (Schnyder et al., 2000; Volanen et al., 2007; Mayer and Thiel, 2014). Therefore, a focus on developing and reinforcing athletes’ and their entourages’ world views (i.e., SoC) is not only relevant for their mental health (Mayer and Thiel, 2014) but also has the potential to support them in their sporting career (e.g., Jindo et al., 2018). However, athletes need to be supported during that process (Henriksen et al., 2020), and thus, the athletes’ entourage needs to initiate the development and reinforcement of the SoC within the COVID-19 pandemic. Therefore, the idea is to strengthen the athletes’ support network’s stress responses so that they can better deal with the uncertainty themselves. At the same time, the aim is to empower the athletes’ support network themselves so that they can then pass on their knowledge and experiences to the athletes they are working with.

In the following, we illustrate coaching techniques aimed at strengthening the SoC in the athletes support network (i.e., SPPs and coaches) during the COVID-19 pandemic. SPPs play a key role in providing psychological support to athletes and also to all other members of the athlete’s support network. At the same time, the SPP is also challenged by the effects of the pandemic. Therefore, our intervention focuses, first of all, on strengthening the SPPs’ SoC so that they, in a second step, can help reinforce and develop their athletes’ SoC.

## SoC Workshop for SPPs

### Aims of the Workshop

The aim of the workshop for SPPs is to enhance their SoC by strengthening their feelings of comprehensibility, manageability, and meaningfulness. This workshop aims at helping them to cope with the unforeseen they experience during the pandemic. Once SPPs have strengthened their SoC, they are better equipped to cope with unforeseen events and can also transfer their knowledge to help athletes and their support network to deal with uncertainty.

### Time, Venue, and Workshop Presenter

The workshop “Coping with Uncertainty” is designed as a 1-day workshop and can be conducted either online or in person. The workshop should be conducted by SPPs with an expertise in salutogenetic approaches. After attending the workshop, participants should be able to conduct their own workshops on SoC with their athletes and the athlete support network.

### Online Versus In-Person Workshop

One might not have the choice of whether to teach the workshop online or in-person, and the format might also have to change at the last minute during the pandemic. In general, online workshops need more preparation time, sometimes face technical difficulties (e.g., poor Internet connection), and have motivational challenges (Rapanta et al., 2020). However, during the pandemic, they are sometimes the only way to conduct a workshop (Carr-Chellman and Duchastel, 2000).

Digital workshop software, such as Zoom, Skype, or BigBlueButton, can be used to conduct the workshop. However, one should be aware that, in some cases, only a professional or licensed version provides access to all of the tools’ features. The tool should be able to subdivide the group into different smaller groups (e.g., breakout rooms or breakout sessions) and provide features, such as whiteboard or shared notes. Furthermore, the tool should enable conducting little surveys. These surveys are useful for assessing the explicit learning objectives and for enhancing the participants’ motivation. It is recommended to thoroughly test the selected tool prior to the workshop both from the role as presenter and the role of participant.

Be sure to conduct a technology check for all participants at least 15 min before the workshop starts so that the participants can become familiar with the online tool and can check their cameras and microphones. We also recommend setting up a few rules regarding manner and communication during online workshops. We suggest the following rules as examples:

• Mute your microphone when you are not speaking.
• Diminish background noises and mute your mobile phone.
• Speak loudly, more clearly, and more slowly than usual.
• Do not use the keyboard when unmuted.
• Use the chat to ask questions or to communicate.

During online workshops, it is recommended to monitor the chat box regularly to ensure that every participant has the opportunity to respond to the presenter’s statements. We also recommend employing additional digital tools during the workshop, like word cloud or mind map tools, to enhance the participants’ motivation. However, it is advisable to only use a limited number of different additional tools.

After the workshop, we suggest leaving the workshop room open for an additional 20 min so that the participants can talk afterward.

In-person workshops foster personal interactions; yet, in times of a pandemic, it is important to pay attention to strict hygiene rules and the obligatory policies aimed at preventing infections. It is, therefore, necessary that you clarify which hygienic standards (e.g., air ventilation, regular disinfecting, and distance between participants) need to be adhered to during the workshop.

In an in-person workshop, it is important to create a communicative space and room for interactions between the workshop participants. Therefore, we recommend setting up several work spaces for single-participant exercises or discussions in smaller groups. Before the workshop starts, participants should have the opportunity to get to know the workshop environment, which can be supported by having some extra welcome time prior to the start of the workshop. Providing snacks and drinks can create a comfortable atmosphere if the hygiene rules allow this.

Furthermore, communication rules should be discussed at the beginning of the workshop. We suggest the following rules as examples:

• Switch off all mobile phones.
• Interference first: Barriers that limit the participants’ focus should be addressed.
• Ask for clarifications, if something is unclear.
• Take notes for raising open questions that could be answered at the end of the workshop.
• Clarify feedback rules.
• Present the rules for how to give feedback.

In-person workshops benefit from using a variety of didactical methods (i.e., single-participant exercises, discussions in smaller groups, and role play) and materials (i.e., flip charts, whiteboards, and moderation cards). A good balance between different didactical methods and a focus on the workshop’s goals should be kept to ensure that the materials fit the workshop activities.

At the end of an in-person workshop, it is helpful to allow extra time for additional or individual questions or personal consultations.

### Target Group and Learning Objectives

The target group are SPPs with an interest in applying new approaches. We expect the participants to be familiar and to follow professional ethical standards (e.g., European Federation of Sport Psychology)^1^. In order to provide the best learning environment, we suggest that the total number of participants does not exceed 15 (Carr-Chellman and Duchastel, 2000). This way, all participants have the opportunity to actively contribute to the workshop, but also to gain knowledge and experience.

This workshop’s aim was to contribute to participants’ explicit and implicit learning processes. Explicit learning refers to learning the actual content (e.g., knowledge and know-how), whereas implicit processes are how content is related to individual perceptions (e.g., experiences, evaluations, meaning, and emotions). Both processes interrelate and support achieving the twofold aim of this workshop (i.e., SoC development in SPPs and transfer to the athlete’s support network). Thus, we define explicit learning (el) objectives to describe what SPPs will know and which skills they will have acquired after attending the workshop. We define implicit learning (il) objectives to describe participants’ ability to reflect on their experiences, values, meaning, and emotions regarding the workshop content.

Explicit learning objectives are as follows:

SPPs are able to

• describe and define the salutogenetic approach.
• identify SoC’s relevance for health promotion and performance enhancement.
• differentiate between the three SoC components.
• identify strategies that can be applied to strengthen the SoC components.
• check and evaluate psychological counseling guidelines and strategies (e.g., Bertollo et al., 2020) published during the COVID-19 pandemic for completeness.
• develop coping strategies based on the salutogenetic approach.
• identify and explain differences in coping strategies according to the SoC components.
• apply the developed strategies to the elite sport setting.
• put themselves in the place of the athlete/the learner and to consider different barriers during the learning process.

Implicit learning objectives are the following:

SPPs are able to

• reflect on their own situation during the COVID-19 pandemic.
• reflect on their own emotional reactions during the COVID-19 pandemic.
• empathize with participants’ emotions and affective states.
• reflect on their own experienced strategies and their effectiveness according to the salutogenetic approach (backspin to introduce self-reflection).
• reflect on their emotional reactions and evaluate different coping strategies.

### Preparation and Material

The following preparation is needed to conduct the workshop:

(1) We suggest preparing slides or handouts (see Supplementary Material) about the salutogenetic approach and current research regarding the crisis of uncertainty due to the COVID-19 pandemic.
(2) Participants are provided with a compendium of guidelines and best practices published by sport psychology organizations during the pandemic. This compendium provides a basic collection of possible salutogenetic approaches that can be applied in their sport psychology practice.
(3) Additional materials to prepare for the workshop parts are:

• Papers.
• Pencils.
• Highlighters.
• Index cards.
• Flip chart marker.
• Flip chart paper.
• Strips (to attach flip charts and sorting cards to a wall).

### Didactics and Organization

Different didactical approaches and organizational forms can be applied in order to ensure that the learning objectives are achieved. The suggested didactical approaches and organizational forms are related to a salutogenetic education (Felbinger, 2010; further description in Section “The Workshop as an Intervention on Participants’ SoC”). Examples are:

• Individual self-reflection about coping during the pandemic.
• Silent reflections on mind maps regarding experiences and coping with uncertainty.
• Lectures about the theory of salutogenesis and related research.
• Brainstorming with the entire group or in small groups, e.g., to collect coping strategies that are based on the salutogenetic model.
• Exercises in small groups, especially evaluation of sport psychological guidelines and strategies developed for the pandemic (e.g., Bertollo et al., 2020) and the development of further coping strategies and techniques.
• Group reflections/flashlights to recapitulate workshop content.
• Group discussions about potentials and barriers of a salutogenetic approach in sport psychology.
• Role plays to apply coping strategies in sport psychological practice.

### Procedure of the Workshop

The workshop comprised four parts:

(1) Knowledge acquisition.
(2) Knowledge transfer.
(3) Knowledge application.
(4) Perspective taking.

In the first part, the workshop leaders present the workshop’s basic theoretical foundation, namely Antonovsky’s model of salutogenesis, and provides a short research overview of similar research on possible crises of uncertainty (e.g., Stambulova et al., 2020). The second part focuses on transferring the knowledge acquired in the first part of the workshop, i.e., participants apply the salutogenetic theory to the applied setting in elite sports. For this, they receive a compendium of possible salutogenetic approaches that can be applied in their sport psychology practice. Building on this compendium, the participants then develop their own toolbox with their most promising coping strategies and techniques to overcome uncertainty.

The workshop should initiate a process of brainstorming and the discussion of different approaches that could be used in sport psychology practice or that could be applied by athletes and their entourage to foster their SoC to cope with uncertainty. Table 1 lists examples of the compendium of possible salutogenetic approaches SPPs can use in their sport psychology practice to develop and to reinforce the SoC, which can be added to their toolbox.

**TABLE 1:** Collection of sense of coherence (SoC) enhancing resources for sport psychology practitioners (SPPs) when coping with uncertainty.

SoC components	Enhancement by following resources	Example
Comprehensibility		
*Explicability*	Information and explanation	Retrieving information from sources of high credibility^a^ Regular communication with others (e.g., team mates and coaches)
*Structure*	Cognitive approaches	Keeping the domestic environment well organized (hygiene, food, fresh air, etc.) supports an athletic lifestyle.^a^ Domestic training might be boring: Brainstorming ideas to keep training at home interesting (e.g., watching TV shows and virtual challenges with team mates)^a^ Regularly repeated psychoeducation to achieve a mental health literacy within the athlete’s support network^b^
*Structure*	Planning and goal setting	Adapting the timetable, not the goals^c^ Setting time slots when to search for information to avoid being overwhelmed by information about the pandemic^a^ Keeping physical training constant during COVID-19 limitations; adaptation of training routines to limitations by training professionals^d^ Developing strategies to reduce too much calorie consumption during a lockdown/training limitations^d^
*Predictability*	Paradoxical intervention	Juxtaposing best- and worst-case scenarios
Manageability	Improving self-efficacy	Developing strategies to incorporate authorities’ restrictions into training and daily life^a^ Teaching relaxation techniques^a,b^
	Self-reflection	Exploration of what keeps athletes effective and disciplined in training^a^ Using self-reflection on strategies that helped you in the past to trigger a pleasant state^a^ Keeping an athletic lifestyle! Relying on all routines, diets, etc., as they still are important and effective to stay healthy, fit, and ready for performance^a^ Technique of circular questioning
	Social networking for support and help	Offering social support and informing when to ask and who to ask for help^a^ Connecting with the athlete’s support network to achieve help to manage upcoming challenges^b,d^ Creating of a professional mental health support network^b^ Including family and friends in the social–emotional support network^b^
Meaningfulness	Satisfaction of needs and motives	Investing time into the neglected but important things that support their goal achievement^c^
	Impact on the athlete’s support network	Trusting in and relating to your social network. You need them; they need you.^a^ Sharing your successful coping strategies with others (in the athlete’s support network). Relating actively to them^a^ Creating a safe environment (social and spatial) where it is accepted to declare mental challenges^b^ Developing a mental health policy^b^ Finding spokespersons for mental health in athletes^b^
	Optimism and positivism	Gratitude exercises and mindfulness-based exercises

*^*a*^Bertollo et al. (2020).*

*^*b*^Henriksen et al. (2020).*

*^*c*^The Open University (2020); https://www.open.edu/openlearn/health-sports-psychology/coronavirus-how-can-athletes-getthrough-period-isolation.*

*^d^Andreato et al. (2020).*

• During the workshop, SPPs can discuss and develop approaches to enhance comprehensibility by strengthening resources that influence individuals’ world views as explicable, structured, and predictable (see Table 1). One approach to make the pandemic and its uncertain consequences explicable could be to only retrieve information from highly credible sources (e.g., national and international health organizations) and to avoid constantly following the news and consulting sources that are not credible (e.g., blogs on the Internet). Adjusting plans and goal setting techniques that change individual training schedules, but not the overall goal, can give structure in this time of uncertainty. As mentioned, predictability is difficult to address in times of uncertainty, and yet, paradoxical interventions, such as contrasting best- and worst-case scenarios, might help individuals.
• SPPs elaborate approaches of manageability to build individuals’ trust in their coping strategies (i.e., resource-oriented coaching approach). Here, the workshop may focus on techniques to improve self-efficacy, self-reflection, social support, and volitional processes. Self-efficacy can be fostered by teaching techniques to adjust affective states or by successfully overcoming training barriers (e.g., integrating governmental restrictions of social distancing into daily training). Self-reflection can enhance self-efficacy, for example by thinking of challenging situations in the past in which successful coping was implemented and how these coping strategies that were successful in the past can be adapted to the current situation.
• Meaningfulness can be defined as accepting the uncertainty of the COVID-19 pandemic as an entirely new challenge for training and competition. Thus, SPPs should focus on holistic approaches when supporting athletes in the challenge to cope with uncertainty. This could be achieved by satisfying individual needs or by creating a supportive social network in which athletes and members of the athlete’s entourage take responsibility for each other. Additionally, investing in mindfulness practice can help individuals to accept the current situation (e.g., mindfulness-based exercises and mindfulness–acceptance–commitment theory; see Stambulova et al., 2020).

These examples are not a complete list, but give an idea of how SPPs can support athletes and their entourage to enhance comprehensiveness, manageability, and meaningfulness when having to cope with uncertainty (see Stambulova et al., 2020 for further approaches).

The third part focuses on knowledge application, during which participants actively apply their new knowledge, for example in role plays, and then discuss possible obstacles they might encounter. In the last part, the participants are asked to reflect on their acquired knowledge and the implicit learning processes that took place during the workshop. The workshop ends with an evaluation of its content and execution and the effect it had on the participants’ SoC. Please see Table 2 and the Supplementary Material for a detailed description of the workshop content and the four parts.

**TABLE 2:** Workshop procedure: schedule, learning objectives, content, didactical methods, and material.

**Workshop phase**	**Learning objectives (el *vs*. il): Participants are able to:**	**Didactical method/organization**
Welcome and introduction	*il*: Create a meaningful relevance by – reflecting on their own situation during the COVID-19 pandemic. – reflecting on their own emotional reactions during the COVID-19 pandemic. – empathizing with participants’ emotions and affective states.	Individual self-reflection about coping during the pandemic Silent reflections on mind maps regarding experiences and coping with uncertainty
Knowledge acquisition	*el*: Enhance comprehensiveness by – describing and defining the salutogenetic approach. – understanding its relevance for health promotion and performance enhancement. – differentiating the three SoC components.	Lectures about the theory of salutogenesis and related research
Knowledge transfer	*el*: Enhance comprehensiveness by – assigning different strategies to the SoC components. – checking psychological counseling guidelines and strategies (e.g., Bertollo et al., 2020) published during the COVID-19 pandemic for completeness.	Exercises in small groups, especially evaluations of psychological counseling guidelines and strategies
Knowledge application	*el*: Enhance comprehensiveness by – developing new coping strategies based on the salutogenetic approach. – identifying and explaining differences in coping strategies according to the SoC components. – discussing the usefulness of the developed strategies for the elite sport context. *il*: Enhance SoC manageability by reflecting their own experienced strategies and their effectiveness according to the salutogenetic approach (backspin to self-reflection during the introduction).	Brainstorming with the entire group or in small groups, e.g., to collect coping strategies according to salutogenesis Exercises in small groups, especially development of further coping strategies Group discussions about potentials and barriers of a salutogenetic approach in sport psychology
Perspective taking	*el*: Enhance manageability by putting themselves in the place of the athlete/learner by considering different barriers during the learning process. *il*: Enhance meaningfulness by reflecting on their own emotional reactions and evaluations of the coping strategies discussed in the workshop.	Role plays to apply coping strategies in sport psychological practice Silent reflections on mind maps regarding experiences and coping with uncertainty
Conclusion		Group reflections/flashlights to recapitulate workshop parts

*el, explicit learning: “comprehensiveness”; *il*, implicit learning: “meaningfulness”, *SoC*, sense of coherence.*

### The Workshop as an Intervention on Participants’ SoC

The workshop is designed in such a way that it simultaneously addresses and achieves the two goals of (1) strengthening SPPs’ SoC and (2) empowering SPPs’ to strengthen their athletes’ SoC. This goal is achieved by dividing the workshop into four parts. The first three parts aim at teaching SPPs not only about the salutogenetic theory but also how to apply it in practice and which obstacles they might encounter. The first three parts (i.e., knowledge acquisition, knowledge transfer, and knowledge application) target SPPs’ comprehensiveness and manageability skills. The last part (i.e., change of perspective) aims at strengthening meaningfulness. By changing perspective, SPPs can identify how meaningful the approach is and also how meaningful it could be for the athletes and their support network in dealing with uncertainty.

#### Connecting Sport Psychology Practice With Salutogenesis Through Meaningfulness

Table 2 presents the workshop’s structure, the learning objectives, and the didactical methods. The last part is crucial for developing a meaningful relationship with the workshop’s learning objectives by reflecting on which sport psychological techniques support athletes in a salutogenetic sense. During the last part of the workshop, the participants change their perspective to that of their athletes and are asked about the barriers their athletes might experience when learning salutogenetic skills. In this part of the workshop, SPPs have to conscientiously reflect on what it means for athletes to apply salutogenetic sport psychology practices. This part of the workshop—connected to Beeler’s (1991) stages of learning—fosters SPPs’ learning about which sport psychological techniques are meaningful for their work with athletes.

According to Beeler (1991), learners (here, SPPs) pass through four different but interrelated stages of learning, namely unconscious incompetence, conscious incompetence, conscious competence, and unconscious competence, and different learning processes occur in each stage. Due to participants’ advanced professional knowledge and experiences, SPPs most probably have already reached Beeler’s fourth stage of unconscious competence with regard to dealing with stress (i.e., highly competent routines in practice that are unconscious for the SPP). However, in order to help athletes to develop and learn new coping strategies, SPPs need to consciously reflect on their own learning process. Making the unconscious competence explicit means that the participants are asked to reflect out loud on their emotional reactions and evaluations throughout the workshop. Whenever participants refer to their implicit processes (e.g., claiming “this strategy works really well for my athletes” with no reference to salutogenesis), the workshop leader should pick up on this and ask participants to reflect out loud so that others can learn from their experiences. Making the implicit processes explicit by asking SPPs to reflect on their current learning processes supports the empowerment of SPPs’ manageability and meaningfulness according to the SoC. This means that SPPs are provided with time during the workshop to emotionally reflect on the coping strategies they like or dislike in their applied work with athletes.

#### Salutogenetic–Education Approach

During the four-part workshop, we follow a salutogenetic–educational approach (Felbinger, 2010) to support the workshop’s psychoeducational aim. Therefore, the workshop uses methods related to the SoC components. In the first three phases, we focus on comprehensibility and manageability. Comprehensibility is enhanced by using cognitive approaches (i.e., lectures), networked learning strategies (i.e., group exercises), a focus on combining knowledge–understanding–developing (i.e., evaluation and further development of COVID-19 pandemic guidelines), hands-on methods (i.e., application of salutogenetic strategies in role plays), and reflections on emotional reactions (i.e., self-reflection at the beginning). Manageability is realized by creating resources *via* self-reflection on comparable situations and one’s own biography (i.e., brainstorming potential salutogenetic approaches out of self-reflection of SPPs’ practical work), as well as social networking and team work (i.e., group exercises). In the fourth phase, we lay a foundation for the development of meaningfulness by reflecting on the workshop’s impact on participants’ SoC regarding coping with uncertainty. We ask about the way the workshop might change their applied work (i.e., flashlights and self-reflection at the end of the workshop). Last but not least, by also asking them to take the athletes’ perspective, we hope to enhance the know-how transfer to the athletes and their support network (i.e., perspective taking).

## Application and Evaluation of the Workshop

The present workshop was conceptualized with already existing sport psychological techniques, and the goal was to rearrange them based on the theoretical framework of salutogenesis. The workshop achieves a twofold educational and psychological aim: on the one hand, workshop participants learn and develop sport psychological approaches they can apply when working with athletes (educational aim). On the other hand, participants develop and reinforce their own SoC (psychological aim). To test whether this twofold aim is successful, we conducted and evaluated a workshop. The target group of the workshop were volleyball coaches and not SPPs. This target group is also highly important for athletes’ welfare and, in addition, also in need of support when major sporting events occur (e.g., 1980s Olympic boycott; Crossman and Lappage, 2002). The workshop content was adapted accordingly.

### Workshop Participants

In a single-day online workshop, *N* = 26 German volleyball coaches participated. Data from 18 coaches (14 males and 4 females) were analyzed. Sixteen coached at local and two at the regional level. They were on average 46 years old (SD = 13 years) and had an average coaching experience of 16 years (SD = 13 years).

### Evaluation Instruments and Procedure

Prior to the workshop, all participants were informed about the content and the goals of the workshop, as well as the evaluation process. Participants volunteered and accepted data assessment, analysis, and anonymous reporting after having received all information and before answering any evaluation questions.

To evaluate the workshop, its effect on SoC, as well as the perception of dealing with uncertainty, participants were asked to answer questionnaires before and after the workshop. For pre- and post-measurement, the questionnaire contained the Sense of Coherence—Leipziger short version (SoC-L9; Schumacher et al., 2000) with three subscales on comprehensibility (two items, *α* = 0.87), manageability (three items, *α* = 0.84), and meaningfulness (four items, *α* = 0.68); a resilience questionnaire (RS-13; Leppert et al., 2008) including 13 items that are answered on a seven-point Likert scale ranging from 1 = *I do not agree* to 7 = *I completely agree* (*α* = 0.73); and a semantic differential to assess coping with uncertainty (instruction: “How do you evaluate your individual coping with the current situation of uncertainty in training and competition?”; semantic differential: 0 = *very poor* to 100 = very good), affective response: valence (0 = *negative* to 100 = *positive*) and arousal (0 = *tense* to 100 = *relaxed*), as well as stress (0 = *stressed* to 100 = *not stressed at all*). Finally, we assessed the workshop quality and the perceived acquired skills by the participants at post-measurement with the Quality Questionnaire for Sport Psychological Coaching (QS-17; Kleinert and Ohlert, 2014). In addition, we asked how likely it is that they will use one of the tools that they have learned during the workshop. Questback was used as a software to collect evaluation data.

### Workshop for Volleyball Coaches

Before the workshop day, the participants were asked to fill out the evaluation questionnaire at pre-measurement. The workshop was adapted to the target group of sport coaches by omitting the fourth phase of the workshop “perspective taking.” Therefore, the workshop had three parts.

(1) Knowledge acquisition: At the beginning, the participants were welcomed and asked to brainstorm current issues when thinking of uncertainty in everyday training. Then, an input session regarding salutogenesis followed.
(2) Knowledge transfer: In a subsequent work phase, the volleyball coaches were asked to brainstorm what they are currently doing with their athletes to address the challenge of uncertainty in training and competition. A second input session regarding SoC enhancers then followed.
(3) Knowledge application: In the third phase, the participants were asked to adapt enhancers of the prior work phase to their own training. Then, the participants discussed these enhancers with a colleague and shared what they want to achieve when interacting with their athletes with the entire group. Finally, the participants reflected on the workshop and were asked to answer the evaluation questionnaire for a second time.

### Data Analysis and Results

The collected data were analyzed using SPSS version 25.0.0. Data were checked for normality and outliers. Two outliers were detected and analyses were run with and without outliers. As the same pattern of results was present, we will report all analyses including outliers. After the descriptive statistics, a multivariate analysis of variances (MANOVA) with repeated measures (pre and post) was calculated to test for changes in the dependent variables (comprehensibility, manageability, meaningfulness, resilience, perception of coping: uncertainty, valence, arousal, and stress).

Descriptive data of all the dependent variables are shown in Table 3. The results show no significant main effect of time [*F*(8, 10) = 1.04, *p* = 0.467, η_*p*_^2^ = 0.454]. Still, for a detailed analysis, the univariate results can be seen in Table 3, showing a decrease in arousal and a tendency for a decrease in uncertainty after the workshop. The descriptive data, even though not overall statistically significant, show that relevant dependent variables can be positively impacted even during a 1-day workshop. Overall, these changes show that the SoC, uncertainty, affect, and stress perception can be positively impacted on a descriptive level, even though coaches had not even applied what they had learned yet. Thus, we would carefully interpret that the workshop provides an added value.

**TABLE 3:** Descriptive statistics of the dependent variables from pre and post workshop, including univariate test results.

	**Pre (SD)**	**Post (SD)**	***p***	**η_p_^2^**
Comprehensibility	5.44 (1.38)	5.69 (1.44)	0.537	0.023
Manageability	5.93 (1.20)	5.98 (0.82)	0.841	0.002
Meaningfulness	5.69 (0.82)	6.00 (0.78)	0.190	0.099
Resilience	75.61 (7.68)	77.28 (6.16)	0.356	0.050
Uncertainty	56.67 (35.60)	68.94 (26.31)	0.084	0.165
Valence	63.61 (27.63)	70.83 (25.17)	0.314	0.059
Arousal	66.17 (25.57)	78.44 (16.08)	0.048	0.210
Stress	75.06 (17.60)	77.11 (15.34)	0.641	0.013

*Comprehensibility, manageability, and meaningfulness can range between 1 and 7. Resilience can range between 13 and 91. Higher scores mean higher values. Uncertainty, valance, arousal, and stress can range between 0 and 100. The higher the number, the more certain, more positive, more relaxed, and less stressed participants feel about the current situation of uncertainty.*

Participants rated the workshop quality on average 3.6 (out of 4, SD = 0.35) and rated the skills they acquired on average as 3 (out of 4, SD = 0.64). On average, the participants believe that they are very likely to use the learned tools in the near future (82%, SD = 24%).

## Outlook

### Practical Perspective on Athletes’ Support

Planning and conducting a workshop to empower one’s SoC in order to better deal with uncertainty in sport psychology practice also is self-help. It allows SPPs to not only empower their athletes but to also reflect on salutogenetic approaches that could be helpful in their own daily life and work. SPPs play a crucial role for athletes when they have to cope with challenging situations (e.g., Papaioannou et al., 2020). The COVID-19 pandemic is such a situation, and it personally affects SPPs as well as athletes and their entire entourage. However, SPPs often do not pay enough attention to their self-care and thereby neglect their mental well-being. SPPs, however, need to be aware of their mental well-being in order to avoid negatively impacting their athletes (Papaioannou et al., 2020). Our workshop provides SPPs with an opportunity to invest in their mental well-being (i.e., to empower their SoC by applying the discussed practices of this workshop to their own lives) as well to further develop tools that they can apply when working with their athletes (i.e., toolbox of salutogenetic strategies and skills) and with other members of the athlete’s entourage (Lim and Pranata, 2020).

Our workshop aims to support SPPs to better prepare for the 2021 Olympics and to better deal with the crisis of uncertainty. The more SPPs participate in workshops to enhance their SoC and to develop salutogenetic tools for their practical work, the more support for athletes to keep on track for Olympics 2021 and beyond can be provided. By that, we follow the idea of creating a mental health network for athletes and their entourage (Henriksen et al., 2020). Throughout the following months, athletes should learn to trust in their SoC by comprehending what is going on, managing their challenges, and finding meaning in coping with uncertainty in order to reduce the crisis’ negative effects (Andreato et al., 2020). We, therefore, suggest that these skills are taught to athletes directly during the crisis (i.e., the toolbox of salutogenetic strategies and skills developed during the workshop). In addition, the salutogenetic approach might also help in dealing with future uncertainties, like having to terminate an athletic career (Stambulova et al., 2020).

Hence, it is necessary to develop SoC-enhancing strategies for SPPs, athletes, coaches, and others to diminish the effects of uncertainty (Taku and Arai, 2020; Mehrsafar et al., 2020). Based on this first collection, the goal of the workshop could be to develop an even larger toolbox of techniques theoretically based on salutogenesis to better cope with uncertainty.

### Theoretical Perspective on Salutogenesis in Peak Performance Sports

Considering salutogenesis as a valuable approach in sport psychology is rather new and advances the research field in two ways. First, the theory of salutogenesis can be integrated as an additional theoretical framework for investigating both peak performance and mental health within the elite athletic community under unforeseen conditions in a normally very planned and controlled environment. Unforeseen changes challenge athletes’ perceptions about being able to perform at a top level despite severe changes in their “normal” environment. In line with existing literature (e.g., Fallby et al., 2006; Mayer and Thiel, 2014; Jindo et al., 2018; Sheykhangafshe et al., 2020), we assume that Antonovsky’s (1993b) salutogenetic model of the SoC can support athletes. Yet, having an impact on the SoC may support not only the athletes but also the other members of the athlete’s support network. Our workshop proposes that SPPs should empower their own SoC before passing their experiences on to their athletes. Thus, we would like to motivate other researchers in the field of sport psychology to investigate the salutogenetic approach and the SoC to derive and (empirically) test sport psychological applications for athletes and other target groups, such as coaches and staff members. Secondly, and connecting to the prior argument, we would expect more research on salutogenesis in elite sports. We theoretically designed a workshop that might support SPPs’ SoC and, in the long term, even athletes’ SoC and thus help them in this crisis of uncertainty. However, evidence for the effectiveness of this approach is lacking. We therefore strongly recommend an evaluation of this workshop with pre and post measurements using standardized psychological questionnaires to assess uncertainty, stress, and the SoC [e.g., Perceived Stress Scale (PSS-10), Reis et al., 2019; Sense of Coherence Scale (SoC-S), Singer and Brähler, 2007]. Similar assessments with athletes who work with SPPs who participated in the workshop should also be conducted.

## Data Availability Statement

The original contributions presented in the study are included in the article/Supplementary Material, further inquiries can be directed to the corresponding author/s.

## Ethics Statement

Ethical review and approval was not required for the study on human participants in accordance with the local legislation and institutional requirements. Written informed consent from the participants was not required to participate in this study in accordance with the national legislation and the institutional requirements.

## Author Contributions

SL and FL came up with the idea and conceptualization. NW and SL planned the workshop. FL and SL conducted and evaluated the workshop. SL wrote the first draft. SL, FL, and NW provided revisions. LK did the literature research and helped with formatting. A-ME proofread the manuscript, contributed to resources, language editing, and supervision. All authors contributed to the article and approved the submitted version.

## Conflict of Interest

The authors declare that the research was conducted in the absence of any commercial or financial relationships that could be construed as a potential conflict of interest.

## Footnotes

1 https://www.fepsac.com/wp-content/uploads/2019/02/ethics.pdf
