# Supplementary material for: Development of a Salutogenesis Workshop for SPPs to Help Them, Their Athletes, and the Athlete’s Entourage Better Cope With Uncertainty During the COVID-19 Pandemic
Source: Front Psychol. 2021 May 21;12:612264. doi: 10.3389/fpsyg.2021.612264 (PMC8177668; doi:10.3389/fpsyg.2021.612264)
Supplement: Supplementary file 1 [file Data_Sheet_1.pdf]

UNIVERSITÄT LEIPZIG

CC BY NC

COPING WITH UNCERTAINTY

A workshop to apply Salutogenesis in sports to cope with a crisis of uncertainty

[1]Sascha Leisterer, [1, 2]Franziska Lautenbach, [1]Nadja Walter, [1]Lara Kronenberg, & [1]Anne-Marie Elbe

[1]Leipzig University (Germany)
Faculty of Sports Science
Institute of Sport Psychology and Sport Pedagogy
Department of Sport Psychology

[2]Humboldt-Universität zu Berlin (Germany)
Faculty of Humanities and Social Sciences
Department of Sport Psychology and Sport Pedagogy
Sport Psychology

CC BY NC

1. **Welcome & Introduction**
2. Knowledge acquisition
3. Knowledge transfer
4. Knowledge application
5. Perspective taking
6. Evaluation & Farewell

Learning objectives

After this workshop you will be able to …

- …describe and define the salutogenetic approach
- …identify SoC's relevance for health promotion and performance enhancement
- …differentiate between the three SoC components
- …identify strategies that can be applied to strengthen the SoC components
- …check and evaluate psychological counseling guidelines and strategies (e.g., Bertollo et al., 2020) published during the COVID19 pandemic for completeness
- …develop coping strategies based on the salutogenetic approach
- …identify and explain differences in coping strategies according to the SoC components
- …apply the developed strategies to the elite sport setting
- …put themselves in the place of the athlete /the learner and to consider different barriers during the learning process

cc BY NC

1. **Welcome & Introduction**
2. Knowledge acquisition
3. Knowledge transfer
4. Knowledge application
5. Perspective taking
6. Evaluation & Farewell

Longitudinal, multifaceted, unpredictive, noncontrolled change-event:

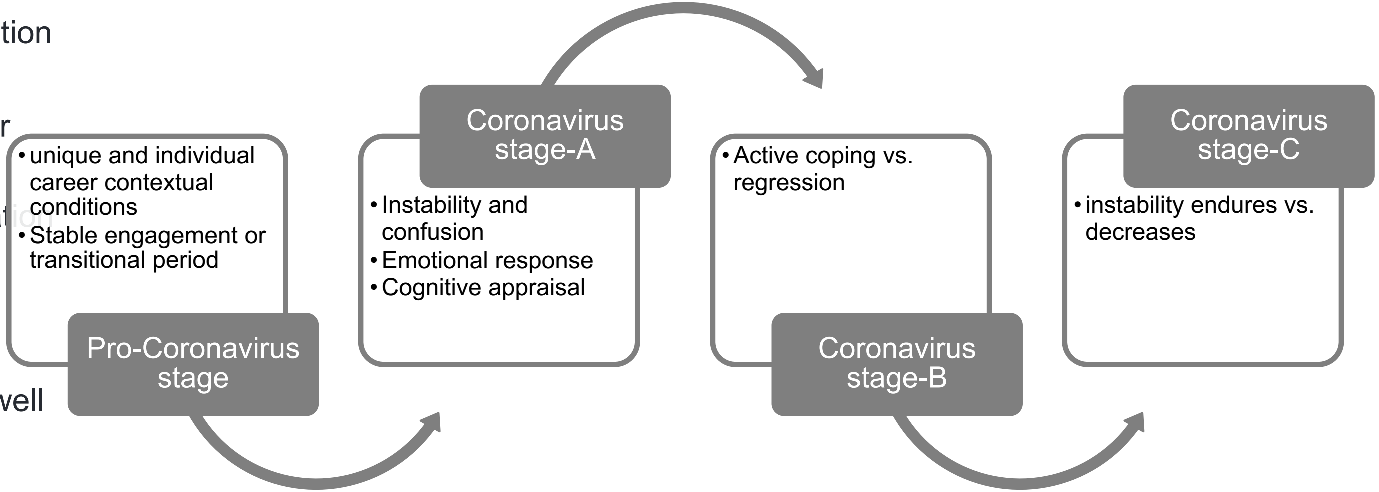

(Samuel et al., 2020)

cc BY NC

1. **Welcome & Introduction**
2. Knowledge acquisition
3. Knowledge transfer
4. Knowledge application
5. Perspective taking
6. Evaluation & Farewell

Athletes and their social support network face an uncertain future due to:

- Stress due to instability and confusion
- Active coping vs. regression
- Unforeseeable instability

Figure 1
Crisis of uncertainty for athletes and their social network, e.g., sport psychological practitioners (SPP).

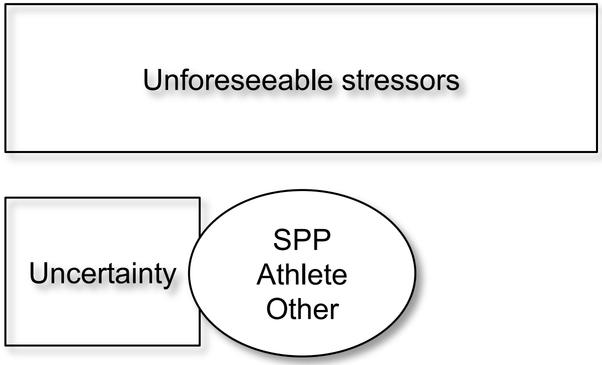

cc BY NC

1. Welcome & Introduction
2. **Knowledge acquisition**
3. Knowledge transfer
4. Knowledge application
5. Perspective taking
6. Evaluation & Farewell

Imagine a river. You jump in and you float with its' flow. Suddenly, a stream pulls you under the surface.

Panic!

Someone at the bank may help you to get out of the water.

But the question is not: "What does the helper need to assist you?"

The salutogenetic question is: "What could have been done before jumping in a river to help yourself?"

(Antonovsky, 1993a)

CC BY NC

1. Welcome & Introduction
2. **Knowledge acquisition**
3. Knowledge transfer
4. Knowledge application
5. Perspective taking
6. Evaluation & Farewell

- **Comprehensibility** refers to the cognitive component of the SoC and encompasses being able to identify stressors correctly and choosing an appropriate coping strategy.
- **Manageability** relates to a feeling of self-efficacy and illustrates a feeling of trust in one's coping skills to effectively manage stressors.
- **Meaningfulness** describes a motivational-emotional drive, which supports individuals in activating a specific coping strategy and in identifying a stressor as an acceptable challenge.

(Antonovsky, 1993b)

Figure 2
SoC support to cope with the unforeseeable.

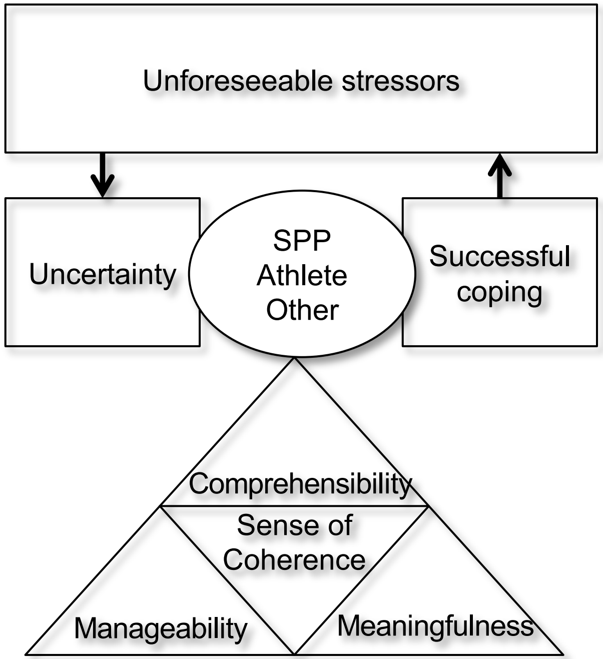

CC BY NC

COVID 19 = career transition barrier

1. Welcome & Introduction
2. **Knowledge acquisition**
3. Knowledge transfer
4. Knowledge application
5. Perspective taking
6. Evaluation & Farewell

- Unanticipated
- Shared
- Uncertainty
- External
- Possibility of a serious illness

(Stambulova etl al., 2020)

CC BY NC

1. Welcome & Introduction
2. **Knowledge acquisitio**
3. Knowledge transfer
4. Knowledge application
5. Perspective taking
6. Evaluation & Farewell

Scenarios for Olympic and Paralympic athletes:

#1 It is a good time for me to retire now.

#2 I can take a pause and strategically prepare for the next Olympic/Paralympic Games

#3 COVID 19 won't stop me to prepare for Tokyo 2020.

(Stambulova etl al., 2020)

cc BY NC

Stress due to the COVID19 pandemic

1. Welcome & Introducti
2. **Knowledge acquisit**
3. Knowledge transfer
4. Knowledge applicatio
5. Perspective taking
6. Evaluation & Farewell

Table 1. Means (and standard deviations) of perceived stress, functional and dysfunctional psychobiosocial states prior to and during COVID-19 pandemic.

	Prior to COVID-19		During COVID-19	
	Women	Men	Women	Men
Perceived stress	13.70 (6.60)	12.10(5.90)	19.50 (5.86)	16.63 (5.58)
Functional psychobiosocial states	2.56 (0.53)	2.65 (0.60)	1.86 (0.69)	2.04 (0.72)
Dysfunctional psychobiosocial states	0.87 (0.46)	0.87 (0.51)	1.46 (0.73)	1.29 (0.74)

Notes: Prior to COVID-19 sample for perceived stress: Women = 926, Men = 1406; prior to COVID-19 sample for functional psychobiosocial states and dysfunctional psychobiosocial states: Women = 70, Men = 181; during COVID-19 sample for perceived stress, functional, and dysfunctional psychobiosocial states: Women = 595, Men = 537. Perceived stress could range between 0 and 40; functional/dysfunctional psychobiosocial states could range between 0 and 4.

(di Fronso et al., 2020)

- Closed sporting facilities (64%)
- Missing social contacts (37%)
- Staying in shape (26%)
- Issues concerning motivation (24%)
- Missing social contacts in the sport context (20%)

(*N* = 95; Lautenbach, Leisterer et al., 2020)

CC BY NC

Stress due to the COVID19 pandemic

1. Welcome & Introductio
2. **Knowledge acquisitic**
3. Knowledge transfer
4. Knowledge application
5. Perspective taking
6. Evaluation & Farewell

Findings. Table 1. *Focus points and themes*

Themes	Focus Points	f	%
Thoughts	Thoughts about the future	12	31.5
	Self-awareness	10	26.3
	Value awareness	9	23.6
	Thoughts about the past	7	18.4
	Total	38	100
Emotions	Anxiety	12	40
	Anger	4	13.3
	Longing	4	13.3
	Depression	3	10
	Sadness	2	6.66
	Stress	2	6.66
	Confidence	1	3.33
	Loneliness	1	3.33
	Fear	1	3.33
	Total	30	100
Management Strategies	Physical Training	17	40.47
	Mental Training	14	33.33
	Take up a hobby	7	16.66
	Trainer support	4	9.52
	Total	42	100
Performance Expectations	Decreased	13	72.22
	Constant	3	16.66
	Increased	2	11.11
	Total	18	100

(*N* = 18; Interview study; Tinganz, 2020)

CC BY NC

1. Welcome & Introduction
2. Knowledge acquisition
3. **Knowledge transfer**
4. Knowledge application
5. Perspective taking
6. Evaluation & Farewell

Comprehensibility
Examples

Regularly repeated psychoeducation to achieve a mental health literacy within the athletic community[4]

Retrieving information from sources of high credibility[2]

Adapting timetables, not goals[1]

CC BY NC

Manageability

Examples

1. Welcome & Introduction
2. Knowledge acquisition
3. **Knowledge transfer**
4. Knowledge application
5. Perspective taking
6. Evaluation & Farewell

Learning how to use relaxation techniques[2, 4]

Exploration of what keeps athletes effective and disciplined in training[2]

Including family and friends as social-emotional support network[4]

cc BY NC

Meaningfulness

Examples

1. Welcome & Introduction
2. Knowledge acquisition
3. **Knowledge transfer**
4. Knowledge application
5. Perspective taking
6. Evaluation & Farewell

Investing time into neglected but important things that support their goal achievement[1]

Developing a mental health policy[4]

cc BY NC

If needed, here is space for your instructions.

1. Welcome & Introduction
2. Knowledge acquisition
3. **Knowledge transfer**
4. Knowledge application
5. Perspective taking
6. Evaluation & Farewell

CC BY NC

If needed, here is space for your instructions.

1. Welcome & Introduction
2. Knowledge acquisition
3. Knowledge transfer
4. **Knowledge application**
5. Perspective taking
6. Evaluation & Farewell

cc BY NC

If needed, here is space for your instructions.

1. Welcome & Introduction
2. Knowledge acquisition
3. Knowledge transfer
4. Knowledge application
5. **Perspective taking**
6. Evaluation & Farewell

CC BY NC

If needed, here is space for your instructions.

1. Welcome & Introduction
2. Knowledge acquisition
3. Knowledge transfer
4. Knowledge application
5. Perspective taking
6. **Evaluation & Farewell**

cc BY NC

Antonovsky, A. (1993a). "Gesundheitsforschung versus Krankheitsforschung [Health Research versus Research of Illness] ," in *Psychosomatische Gesundheit*, ed. A. Franke and M. Broda (Tübingen: dgvt-Verlag), 3-14.

Antonovsky, A. (1993b). The structure and properties of the coherence sense. *SoC. Sci. Med.* 36:6, 725-733.

Di Fronso, S., Costa, S., Montesano, C., Di Gruttola, F.,Giorgio Ciofi, E., Morgilli, L., Robazza, C., and Bertollo, M. (2020). The effects of COVID-19 pandemic on perceived stress and psychobiosocial states in Italian athletes. *Int. J. Sport Exerc. Psychol.* doi: 10.1080/1612197X.2020.1802612

Lautenbach, F., Leisterer, S., Walter, N., Kronenberg, L., Manges, T., Leis, O., Pelikan, V., Gebhardt, S., and Elbe, Anne-Marie. (submitted). Athletes' motivation to exercise, stress, and coping during the corona crisis. *Front. Psychol.*

Samuel, R.D., Tenenbaum, G., and Galily, Y. (2020). The 2020 Coronavirus pandemic as a change event in sport performers' careers: Conceptual and applied practice considerations. *Front. Psychol.* 11:567966. doi: 10.3389/fpsyg.2020.567966

Stambulova, N. (2011). The mobilization model of counseling athletes in crisis-transitions: An educational intervention tool. *J. Sport Psychol. Action* 2:3. doi: 10.1080/21520704.2011.598223

Tingaz, E.O. (2020). The psychological impact of COVID-19 pandemic on elite athletes, management strategies and post-pandemic performance expectations: A semi structured interview study. *Int. J. Educ. Res. Inn.* 15, 73-81. doi: 10.46661/ijeri.4863

CC BY NC

UNIVERSITÄT LEIPZIG

Thank you very much for your participation, contributions and idea exchanges!

GOOD BYE!

Stay resilient and be well!

Contacts:

Sascha Leisterer & Dr. Franziska Lautenbach

sascha.leisterer@uni-leipzig.de | franziska.lautenbach@uni-leipzig.de

Leipzig University (Germany)
Faculty of Sports Science | Institute of Sport Psychology and Sport Pedagogy | Department of Sport Psychology
Jahnallee 59, 04109 Leipzig, Germany

http://www.spowi.uni-leipzig.de/fakultaet/institute-fachgebiete/psychpaed/sportpsychologie/
