# Supplementary material for: Development of a Salutogenesis Workshop for SPPs to Help Them, Their Athletes, and the Athlete’s Entourage Better Cope With Uncertainty During the COVID-19 Pandemic
Source: Front Psychol. 2021 May 21;12:612264. doi: 10.3389/fpsyg.2021.612264 (PMC8177668; doi:10.3389/fpsyg.2021.612264)
Supplement: Supplementary file 2 [file Data_Sheet_2.pdf]

Supplement A

Workshop procedure: Schedule, learning objectives, content, didactical methods, and material.

Time (approx.)<br><br>(min)	Workshop phase	Learning objectives (el vs. il)<br>Participants are able to …	Content	Didactical method/ organization	Material
Prior to workshop	Preparation		For Workshop guidance: Inform participants about workshop framework, provide material, and collect questions beforehand		Literature, guidelines, slides
30 min	Welcome & introduction	il: …create a meaningful relevance by…<br>- …reflecting their own situation during the COVID19 pandemic.<br>- …reflecting their own emotional reactions during the COVID19 pandemic.<br>- …empathizing with participants' emotions and affective states.	- Clarification/repetition of workshop schedule and learning objectives<br>- Introduction of participants by answering the questions: "How is work during the COVID19-pandemic?", "How do you personally deal with the COVID19-pandemic?"	Individual self-reflection about coping during the pandemic<br><br>Silent reflections on mind-maps regarding experiences and coping with uncertainty	For reflections on mind-maps: Papers, pencils
60 min	Knowledge acquisition	el: …enhance comprehensiveness by…<br>- …describing and defining the salutogenetic approach.<br>- …understanding its relevance for health promotion and performance enhancement.<br>- …differentiating the three SoC	- Definition of: stress and stressors, SoC, resources<br>- Introduction of salutogenetic model and SoC elements<br>- Overview: Uncertainty research in sports	Lectures about the theory of Salutogenesis and related research	PPT slides

		components			
20 min	Break				
40 min	Knowledge transfer	el: …enhance comprehensiveness by…<br>- …assigning different strategies to the SoC components.<br>- …checking psychological counseling guidelines and strategies (e.g., Bertollo et al., 2020) published during the COVID19 pandemic for completeness.	- Evaluation of trategies to cope with stressors<br>- Evaluation of psychological counseling guidelines to cope with the COVID19-pandemic	Exercises in small groups, especially evaluations of psychological counseling guidelines and strategies	Collection of psychological counseling guidelines as handouts<br><br>For evaluation: Index cards, Flip Chart markers, Flip Chart paper
60 min	Knowledge application	el: …enhance comprehensiveness by…<br>- …developing new coping strategies based on to the salutogenetic approach.<br>- …identifying and explaining differences in coping strategies according to the SoC components.<br>- …discussing the usefulness of the developed strategies for the elite sport context.<br><br>il: …enhance SoC manageability by reflecting their own experienced strategies and their effectiveness according to the salutogenetic approach (backspin to self-reflection during the introduction).	- Development and discussion regarding new coping strategies	Brainstorming with the entire group or in small groups, e.g., to collect coping strategies according to Salutogenesis<br><br>Exercises in small groups, especially development of further coping strategies<br><br>Group discussions about potentials and barriers of a salutogenetic approach in sport psychology	For brainstorming: Index cards, Flip Chart markers, Flip Chart paper<br><br>For discussion (collecting potentials and barriers): Flip Chart paper, Flip Chart markers, strips
60 min	Break				
90 min	Perspective taking	el: …enhance manageability by …<br>- …putting themselves in the place		Role plays to apply coping strategies in sport	

	("manageability")	of the athlete /learner.<br>- …by considering different barriers during the learning process.<br><br>il: …enhance meaningfulness by<br>- …reflecting on their own emotional reactions and evaluations of the coping strategies discussed in the workshop.<br><br>il: …enhance meaningfulness by<br>- …reflecting on their own emotional reactions and evaluations of the coping strategies discussed in the workshop.		psychological practice<br><br>Silent reflections on mind-maps regarding experiences and coping with uncertainty	Mind-maps from the beginning, pencils<br><br>For reflections on mind-maps: Papers, pencils
30 min	Conclusion		- Feedback<br>- Evaluation	Group reflections/flashlights to recapitulate workshop parts	Any object for flashlight recapitulation: ball, card, …<br><br>Feedback / evaluation questionnaires

Notes: el = explicit learning: "comprehensiveness"; il = implicit learning: "meaningfulness", SoC = Sense of Coherence
